# Dissecting the Spectrum of Rare BRAF Mutations in Melanoma: A Nation‐Wide Study by the Italian Melanoma Intergroup (IMI)

**DOI:** 10.1111/pcmr.70087

**Published:** 2026-04-19

**Authors:** Bruna Dalmasso, Maria Chiara Scaini, Laura Cendron, Monica Rodolfo, Ilaria Mattavelli, Elena Tamborini, Francesca Collina, Gerardo Ferrara, Gabriele Madonna, Carlo Cota, Elisa Melucci, Stefania Tommasi, Martina Ubaldi, Roberta Depenni, Andrea Carugno, Riccardo Marconcini, Laura Orgiano, Maurizio Lombardo, Simona Sola, Cristina Pellegrini, Francesca Castiglione, Matteo Gasparotto, Alireza Jorkesh, Stefania Pellegrini, Edoardo Raposio, Andrea Boutros, Enrica Teresa Tanda, Michele Guida, Pietro Quaglino, Giuseppe Palmieri, Daniela Massi, Paolo Antonio Ascierto, Francesco Spagnolo, Mario Mandalà, Chiara Menin, Paola Ghiorzo, Lorenza Pastorino, Italian Melanoma Intergroup, Lorenza Di Guardo, Roberto Patuzzo, Maria Colombino, Simone Ribero, Rebecca Senetta, Gabriele Roccuzzo, Maria Concetta Fargnoli, Cesare Massone, Maria Lentini, Giuseppe Giuffrè, Teresa Troiani, Denise Morotti, Miriam Paone

**Affiliations:** ^1^ Cancer Genetics IRCCS Azienda Ospedaliera Metropolitana, Ospedale Policlinico San Martino Genoa Italy; ^2^ Immunology and Molecular Oncology Unit Veneto Institute of Oncology—IOV IRCCS Padua Italy; ^3^ Department of Biology University of Padua Padua Italy; ^4^ Unit of Translational Immunology, Department of Experimental Oncology Fondazione IRCCS Istituto Nazionale dei Tumori Milan Italy; ^5^ Melanoma Surgical Unit Fondazione IRCCS Istituto Nazionale dei Tumori Milan Italy; ^6^ Department of Diagnostic Innovation Fondazione IRCCS Istituto Nazionale dei Tumori Milan Italy; ^7^ Pathology Unit, Istituto Nazionale Tumori IRCCS—Fondazione G. Pascale Naples Italy; ^8^ Melanoma, Cancer Immunotherapy and Innovative Therapies Unit Istituto Nazionale Tumori IRCCS Fondazione G. Pascale Napoli Italy; ^9^ Dermatopathology Unit San Gallicano Dermatological Institute IRCCS Rome Italy; ^10^ Pathology Unit, IRCCS Regina Elena National Cancer Institute Rome Italy; ^11^ Molecular Diagnostics and Pharmacogenetics Unit IRCCS Istituto Tumori ‘Giovanni Paolo II’ Bari Italy; ^12^ Unit of Medical Oncology University of Perugia Perugia Italy; ^13^ Department of Oncology and Hematology University of Modena and Reggio Emilia Modena Italy; ^14^ Department of Medicine and Surgery University of Insubria Varese Italy; ^15^ Medical Oncology Unit Azienda Ospedaliero‐Universitaria Pisana Pisa Italy; ^16^ Department of Medical Oncology University of Cagliari Cagliari Italy; ^17^ Division of Dermatology, Department of Medicine and Surgery, Ospedale di Circolo e Fondazione Macchi ASST Dei Sette Laghi Varese Italy; ^18^ Surgical Pathology Galliera Hospital Genoa Italy; ^19^ Department of Biotechnological and Applied Clinical Sciences University of L'Aquila L'Aquila Italy; ^20^ Histopathology and Molecular Diagnostics Careggi University Hospital Florence Italy; ^21^ Section of Pathology, Department of Health Sciences University of Florence Florence Italy; ^22^ Department of Translational Brain Research, Central Institute of Mental Health, Medical Faculty Mannheim Heidelberg University Mannheim Germany; ^23^ Plastic Surgery Division, Department of Surgical Sciences and Integrated Diagnostics (DISC) University of Genoa Genova Italy; ^24^ Skin Cancer Unit, Azienda Ospedaliera Metropolitana Ospedale Policlinico San Martino Genoa Italy; ^25^ Department of Internal Medicine and Medical Specialties University of Genoa Genoa Italy; ^26^ Rare Tumors and Melanoma Unit IRCCS Istituto Tumori Giovanni Paolo II Bari Italy; ^27^ Dermatology Clinic, Department of Medical Sciences University of Turin Turin Italy; ^28^ Unit of Cancer Genetics, Immuno‐Oncology & Targeted Cancer Biotherapies, IRGB‐CNR University of Sassari Sassari Italy; ^29^ Medical Oncology Unit, Santa Maria Misericordia Hospital University of Perugia Perugia Italy; ^30^ Italian Melanoma Intergroup (IMI) Genova Italy

## Abstract

Non‐V600E/K BRAF mutations have been reported in melanoma, but data on their clinical relevance are conflicting. This study investigated the distribution, prognostic role, and functional impact of rare BRAF mutations in melanoma. We retrospectively assessed frequency, response to therapy and outcome of rare BRAF mutations compared to V600E/K in cases from 19 Italian Melanoma group (IMI) centers. 258/14,081 samples (1.8%) harbored rare BRAF mutations, 40% encompassing codon 600. Overall and progression‐free survival (OS, PFS) following target therapy with BRAF/MEK inhibitors were comparable to V600E/K mutant melanoma (HR = 0.85 and 0.89, *p* > 0.1). Response to target therapy was lower, albeit not significantly, in rare BRAF mutant melanomas compared to V600E/K (48% vs. 66%, *p* > 0.05). OS, PFS, and objective response in cases treated with immunotherapy were unaffected by BRAF status. Molecular dynamics simulation assessing whether selected BRAF variants affected BRAF structure similarly to V600E showed variable degrees of destabilization towards constitutive protein activation, particularly for mutations encompassing codons 599–601. These results indicate that rare BRAF mutations can modify BRAF kinase activity including a subset of mutations outside but close to codon 600. Molecular approaches able to detect rare BRAF mutations could identify additional melanoma cases eligible for therapies with BRAF/MEK inhibitors.

## Research Highlights

- Almost 2% of melanomas in Italy harbor rare V600 and non‐V600 BRAF mutations, with survival and response to targeted therapy comparable to V600E/K.
- In‐silico modeling shows that specific rare BRAF mutations can have different effects on the functional structure of the protein, depending on their location and biochemical characteristics.

## Graphical abstract

In a large multicentre Italian cohort (14,081 melanoma cases from 19 centres), rare BRAF mutations (~2%) showed survival and response to targeted therapy comparable to V600E/K. Functional modeling indicates that variants, particularly near codon 600, can induce constitutive activation, supporting expanded BRAF genotyping to identify additional patients eligible for BRAF/MEK inhibitor therapy.

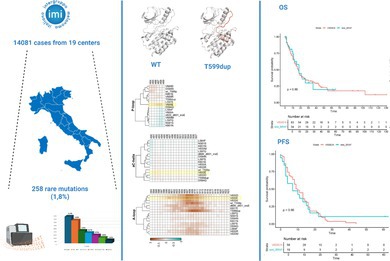

## 1 Introduction

Immunotherapy and target therapy with BRAF/MEK inhibitors (BRAFi/MEKi) brought radical changes to melanoma treatment (Davies et al. 2002; Hodi et al. 2010), with a marked increase in overall survival (OS) (Flaherty et al. 2012; Wolchok et al. 2022). Current guidelines recommend immunotherapy as the preferred first‐line treatment (Amaral et al. 2025). After progression, or when first‐line immunotherapy is contraindicated, unresectable and/or metastatic BRAF mutant (V600E or V600K) cutaneous melanoma can be treated with BRAFi/MEKi (Sondak et al. 2024). Consequently, somatic BRAF genotyping is pivotal for the individualized treatment of advanced melanoma (Barbour et al. 2014; Vanni et al. 2020). The most common BRAF mutations in melanoma are V600E and V600K, but non‐V600E/K BRAF variants have been reported, albeit less frequently. These variants, including K601E, L597Q, and D594G, add to the complexity of choosing a treatment strategy for melanoma. However, clinical trials that led to the approval of target therapy only included cases with BRAF V600E/K mutant melanoma (Bowyer et al. 2014; Comito et al. 2022; Marconcini et al. 2017; Moiseyenko et al. 2019; Nebhan et al. 2021; Rogiers et al. 2017, 2019), whereas evidence regarding other BRAF mutations is less robust.

The potential actionability of rare BRAF mutations was first evaluated by Menzer et al. who analyzed a retrospective case series of 96 patients carrying rare variants (58 at codon 600 and 38 Non‐V600), showing that the BRAFi/MEKi combination appeared to be the best regimen for mutations at both codon V600 and non‐V600. However, the sample size was small and heterogeneous (Menzer et al. 2019).

More recently, a multicenter French study conducted on 856 melanoma cases, including 51 with rare BRAF mutations, found that rare BRAF variants involving codon 600 conferred sensitivity to MAPK inhibitors similar to that of V600E/K, whereas clinical benefits seemed lower for variants outside codon 600 (Girod et al. 2022).

**FIGURE 1:**
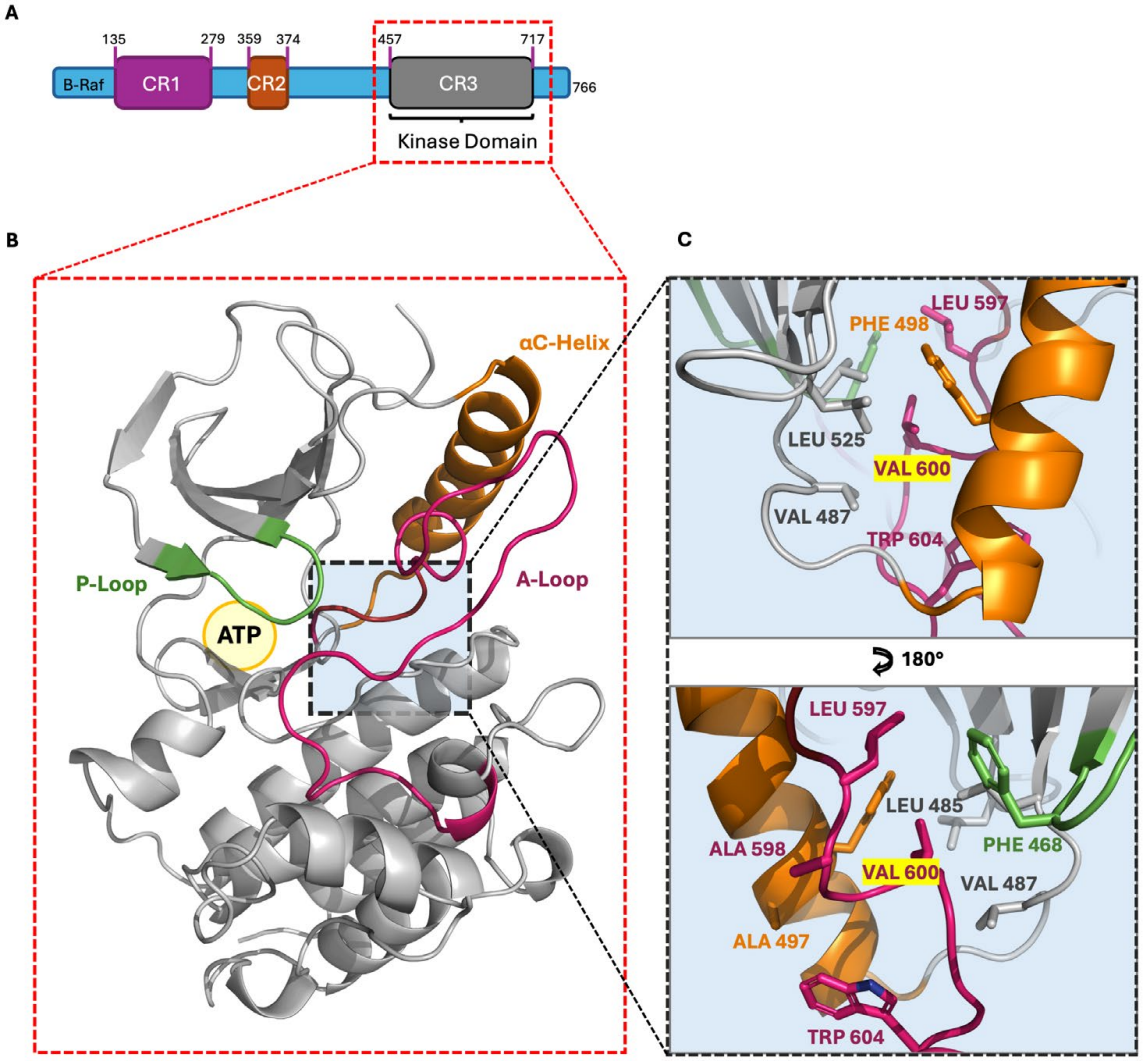
Panel A: Schematic representation of BRAF protein and its domains. The numbers indicate the start and end points of each domain: CR1 spans from residues 135 to 279, CR2 from 359 to 374, and CR3 from 457 to 717. Panel B: Close‐up view of the CR3 domain, which is the kinase domain of BRAF. Three key regions are highlighted: The P‐loop (green), the activation loop (A‐loop, hot pink), and the αC helix (orange). The DFG motif is also indicated within a boxed region. Panel C: Focus on the Val600 residue and the surrounding amino acids. The names of the amino acids are color‐coded according to their respective domains, with Val600 highlighted in yellow. The two panels depict the same zoomed‐in region, with distinct details, emphasized by a 180‐degree rotation.

Other in‐vitro studies, case reports, and small case series on non‐V600E/K mutant melanoma have reported clinical benefits from targeted therapy, especially combined BRAFi/MEKi (Keller et al. 2021). On the other hand, other classes of molecules have been developed to target BRAF with non‐V600E/K variants exploiting their effect on the kinase activity and oligomerization (Brummer and McInnes 2020). Under physiological conditions, inactive BRAF exists in a self‐inhibited conformation, and the interaction with Ras promotes its autophosphorylation of tyrosine 599 and serine 602 within the activation loop (A‐loop, residues 593–623). These phosphorylations induce conformational changes that lead to kinase activation and, ultimately, MEK phosphorylation (Lavoie and Therrien 2015). BRAF kinase activation and ATP binding imply the involvement of three major structural elements within the same domain, that are the activation loop (A‐loop), the P‐loop (464–469), which participates in coordinating the ATP γ‐phosphate group, and the αC helix (490–507) (Figure 1), which shifts from an outward to an inward position, pointing toward the catalytic site in the active conformation (Köhler and Brummer 2016). Therefore, amino acids belonging to all the mentioned sites participate, to varying degrees, in the mechanism of kinase activity, shift from closed (inactive) to open (active) state, independence from dimerization, and protein stabilization. In this scenario, understanding which amino acid change can constitutively activate BRAF, making it a driver of melanoma, could therefore help to understand which other mutations besides V600E/K should be targeted with BRAFi/MEKi for a presumed greater likelihood of success.

This study aimed to determine the frequency of non‐canonical variants in exon 15 of the BRAF gene (rare BRAF mutations) and assess their clinical prognostic impact by retrieving clinical information from 19 centers within the Italian Melanoma Intergroup (IMI).

To enhance the classification of the variants and assess their actionability, molecular dynamics simulations were performed to explore whether some rare BRAF variants identified in our cohort lead to destabilizing effects on BRAF structure similarly to V600E.

## 2 Materials and Methods

### 2.1 Data Collection and Molecular Analysis

We included a retrospective series of melanoma cases with non‐V600E/K BRAF variants collected from existing medical records and databases by the participating centers from June 2012 to November 2023. Inclusion criteria were: metastatic disease, BRAF exon 15 genotyping (either in the primary tumor or metastasis) using any technique.

Information requested included the characteristics of the primary melanoma (age of diagnosis, histotype, mitotic rate, ulceration, and regression), site(s) of metastasis, therapy type and setting, and last follow‐up. For molecular analyses, the required data were the type of lesions analyzed (primary or metastatic melanoma, lymph node, skin/soft tissue, or visceral), the percentage of neoplastic cells in the lesion, and the routine diagnostic molecular testing method used (Pyrosequencing, Sanger, Next‐generation Sequencing [NGS], RT‐PCR, MALDI‐TOF, etc.). BRAF mutations are commonly classified into three categories based on their impact on kinase activity and their dependence on RAS signaling (Sahin and Klostergaard 2021; Wan et al. 2004). This classification, however, is limited to V600 mutations and mutations outside codon 600 for which functional data exist and could not be applied to all mutations found in our cohort. Therefore, BRAF mutations were categorized based on AMP/ASCO/CAP classification guidelines (Li et al. 2017). Each participating center was also requested to report the total number of samples analyzed and V600E/K variants identified. From two centers, we were also able to collect information on clinical characteristics, treatment, and survival in cases with V600E/K mutations recruited at the same time in order to compare them to cases with rare BRAF mutations.

The local Ethics Committee approved the study protocol (CER Liguria, 198/2023), which was conducted according to the Helsinki declaration guidelines and all applicable local regulations.

### 2.2 Molecular Dynamics Simulations

Models of wt and variants of BRAF kinase domain spanning residues 447–722 were built based on the crystal structure of BRAF trapped in the inactive state (PDB ID 6PP9) (Eswar 2003; Park et al. 2019). For this analysis, we selected rare BRAF mutations found in melanomas from cases treated with target therapy in our study cohort, or variants already functionally studied to be used as controls.

Variants were introduced with PyMol v2.3.5 mutation wizard (Brunger and Wells 2009) so that the starting point of all mutations and post‐translational modifications was comparable. All‐atom MD simulations were performed in triplicates, as already described in Chinellato et al. (2023). Briefly, simulations were run with Gromacs 2022.3 using the Charmm36‐jul2021 force field (Abraham et al. 2015; Berendsen et al. 1995; Soteras Gutiérrez et al. 2016; Vanommeslaeghe et al. 2010). Models were solvated with the TIP3 water model in a triclinic box, with a minimum distance of 1 nm between the protein and the box surface; 0.15 M NaCl was added to simulate physiological ionic strength and neutralize the system. System energy was minimized by 5000 steps of steepest descent energy minimization, with a 1000 kJ mol^−1^ nm^−1^ tolerance. Subsequently, a 200 ps NVT MD simulation was used to heat the system from 0 to 100 K with restraints lowered to 400 kJ mol^−1^ nm^−2^. Then, the system was heated up to 310 K in 400 ps during an NPT simulation with further lowered restraint (200 kJ mol^−1^ nm^−2^).

Finally, the system was equilibrated during an NPT simulation for 1 ns with backbone restraints lowered to 50 kJ mol^−1^ nm^−2^. All restraints were removed for the 1 μs production run. The V‐rescale thermostat was used to equilibrate the temperature, whereas the C‐rescale barostat was used to control the pressure (Bernetti and Bussi 2020; Bussi et al. 2007). Newton's equation of motion was integrated using a leapfrog algorithm with a 2‐fs time step. The particle mesh Ewald (PME) method was used to compute the long‐range electrostatic forces, and H‐bonds were constrained using the LINCS algorithm (Darden et al. 1993; Essmann et al. 1995).

The convergence of the final MD trajectories was determined by evaluating the cosine content of the protein backbone RMSD (root mean square deviation) versus time plot. The RMSF of convergent simulations was then compared to the wt and the V600E mutant to assess alteration in protein flexibility. Simulations were performed on models of the protein with an inactive structure as well as with the phosphorylated BRAF T599p, the reference variant BRAF V600E, and the rare variants collected in this study.

### 2.3 Statistical Analysis

To assess the association of BRAF status with clinical‐pathological characteristics, we used the Wilcoxon rank sum test (for numerical variables) and chi‐square test or Fisher exact test (for categorical variables). Using a binomial test, we compared the overall response rate (ORR) of cases with rare BRAF mutant melanomas with an expected rate, calculated as the weighted median of ORR from phase III clinical trials and real‐world studies. The studies and the expected rate are reported in a previous publication (Spagnolo et al. 2021). To assess differences in response to therapy between non‐V600E/K and V600E/K in our study cohort, we used a chi‐square test or Fisher exact test. When control for a confounding variable was needed, we also used the Cochran–Mantel–Haenszel test. To assess overall survival (OS), events were defined as death by any cause, and survival time was calculated as months from the start of therapy to death or censoring. For progression‐free survival (PFS), events were defined as melanoma progression or death by any cause, whichever occurred first, whereas survival time was defined as months between the start of therapy to disease progression or censoring. Cases with no event were censored at the last follow‐up date. We analyzed univariate OS and PFS with the Kaplan–Meier estimator and corresponding Log‐rank derived *p*‐value. Hazard ratios and corresponding 95% confidence intervals were obtained for both univariate and multivariate survival analysis using cox‐proportional hazard regression models. All tests were two‐sided, and a *p*‐value of 0.05 was used to determine statistical significance. Statistical analyses were conducted and plots were generated within the R computational environment (R version 4.4.3), using the R and Bioconductor packages rio, tidyverse, gtsummary, rstatix, janitor, flextable, survival, survminer, survcomp (Chan et al. 2023; R Core Team 2025; Schröder et al. 2011; Sjoberg et al. 2021; Therneau 2024; Wickham et al. 2019).

## 3 Results

### 3.1 Type and Frequency of Rare BRAF Mutations and Patients' Characteristics

Out of 14,081 melanoma cases collected from 19 IMI centers, we retrieved 6131 and 282 cases with BRAF V600E/K and rare non‐V600E/K melanoma, respectively. After filtering out samples with BRAF mutations outside exon 15 (*N* = 9), variants of unknown significance (VUS, *N* = 4), and with concurring V600E/K mutations (*N* = 11), 258 cases were evaluated (Table S1). Cases with non‐V600E/K BRAF mutant melanoma (rare_BRAF group) were older at diagnosis than those in the BRAF V600E/K subset with available clinical data (*N* = 150) (median age 65.5 vs. 59 years, respectively, *p* < 0.001), and the death rate was lower in rare_BRAF (31% vs. 57% in V600E/K, *p* < 0.001), whereas sex and melanoma histotype did not significantly vary. An overview of clinical characteristics of cases included in this study is reported in Table S2.

The overall detection rate of rare BRAF mutations was 1.8% (258/14,081 samples), ranging from 0.31% to 6.35% among the different centers, probably due to the different case numbers and depending on the molecular analysis technique used by each center. Notably, rare BRAF mutations accounted for 4% of all BRAF mutations identified (258 out of 6389 samples) (Table S1 and Figure S1). Rare BRAF mutations located at codon V600 represented 40.3% of all rare mutations (Table 1). The most frequent rare BRAF mutation identified was V600R (24.03%), followed by K601E (21.71%) and V600D (7.36%). Other mutations T599dup and L597S were found in 6.20% and 5.43% of cases, respectively.

**TABLE 1:** Type and frequency of rare BRAF mutations found within (V600_other) and outside (Non_V600) codon 600 in the study cohort.

V600_other	*N*	% (*N* = 258)	Non V600	*N*	% (*N* = 258)
V600_K601delinsE	8	3.10	N581I,S	4	1.55
V600_K601delinsEQ	1	0.39	L584F	6	2.33
V600A	1	0.39	E586K,G,V	5	1.94
V600D	19	7.36	L588F	1	0.39
V600M	3	1.16	G593D	1	0.39
V600R	62	24.03	D594E,G,N,Y	20	7.75
V600_W604delinsS	2	0.78	G596C,D,R	5	1.94
V600D/R	6	2.33	L597E,K,Q,R	13	5.04
V600M/R	2	0.78	L597S	14	5.43
			A598_T599insV	1	0.39
			T599_V600insT	1	0.39
			T599dup	16	6.20
			T599I	2	0.78
			K601E	56	21.71
			K601D,N,R	5	1.94
			R603G	1	0.39
			W604G	1	0.39
			Q609X	1	0.39
			E611K	1	0.39
Total no.	104	40.31%		154	59.68%

### 3.2 Rare BRAF Mutation Detection Rate According to Molecular Analysis Technique

Used by 16 out of 19 centers, Next‐Generation Sequencing (NGS) was the most commonly employed and effective method for identifying rare BRAF variants in terms of mutation frequency, achieving a detection rate of 2.95% (106/3594), whereas Sanger sequencing was used by 6 centers with a slightly lower detection rate (2.15%, 90/4196). Detection rate by pyrosequencing was 1.87% (9/482), consistent with that of Real‐Time PCR (1.32%, 26/1977), whereas other methods such as PNA‐based techniques, MALDI‐TOF, and EasyPGX, achieved lower detection rates (Figure S2). Notably, none of the IMI centers relied on a single testing method. In routine practice, and largely due to cost considerations, many centers initially applied assays specifically targeting the more prevalent V600 mutations. Samples identified as wild‐type by this first‐line approach were subsequently subjected to more comprehensive molecular analyses, such as NGS or Sanger sequencing, to enable the detection of rare BRAF variants.

### 3.3 Response to Target Therapy

Forty cases with melanoma harboring rare BRAF variants were treated with target therapy as first or second line therapy. ORR in the rare BRAF group was lower (48%) compared to that expected from phase III studies (66%) and real‐world studies (57%) focused on V600E/K mutant melanoma, but these differences were not significant (*p* > 0.05).

We then compared rare_BRAF to a subset of V600E/K included in this study with available information on treatment from two participating centers (*N* = 89), obtaining comparable results (ORR 48% vs. 66%, *p* > 0.1). Notably, V600K ORR was higher than V600E (82% vs. 64%), although the difference was not significant. These results remained consistent after adjusting for therapy setting. Clinical characteristics of rare_BRAF and V600E/K treated with target therapy are shown in Table 2. Detailes on differences between rare_BRAF, V600E and V600K, with pairwise comparisons, is shown in Table S3.

**TABLE 2:** Clinical characteristics of cases treated with target therapy.

Variable	*N*	V600E/K (*N* = 89)	rare_BRAF (*N* = 40)	*p* ^c^
Age (years)^a^	125	62.0 (50.0, 72.0)	64.5 (56.0, 74.0)	0.3
Missing		2	2	
Sex^b^				
F	129	29/89 (33%)	12/40 (30%)	0.8
M		60/89 (67%)	28 / 40 (70%)	
Histotype^b^				
SSM	49	9/30 (30%)	12/19 (63%)	0.078
NM		17/30 (57%)	6/19 (32%)	
LMM		0/30 (0%)	0/19 (0%)	
ALM		0/30 (0%)	0/19 (0%)	
Other		4/30 (13%)	1/19 (5.3%)	
Missing		59	21	
OS status^b^				
ALIVE	126	24/87 (28%)	19/39 (49%)	0.021
DEAD		63/87 (72%)	20/39 (51%)	
Missing		2	1	
Best response—First/second line^b^				
CR	108	15/85 (18%)	3/23 (13%)	0.4
PR		41/85 (48%)	8/23 (35%)	
SD		10/85 (12%)	5/23 (22%)	
PD		19/85 (22%)	7/23 (30%)	
Missing		4	17	
ORR—First/second line^b^	108	56/85 (66%)	11/23 (48%)	0.11
Missing		4	17	
Median OS	120	15.0 (6.0, 33.0)	11.0 (5.0, 22.0)	0.3
Missing		4	5	
Median PFS	117	10.5 (4.0, 21.0)	10.0 (3.0, 16.0)	0.5
Missing		7	5	

Abbreviations: CR, complete response; F, female; M, male; NM, nodular melanoma; ORR, overall response rate; OS, overall survival; PD, progressive disease; PFS, progression‐free survival; PR, partial response; SD, stable disease; SSM, superficial spreading melanoma.

^a^ Median (IQR).

^b^ *n*/*N* (%).

^c^ Wilcoxon rank sum test; Pearson's Chi‐squared test; Fisher's exact test.

Objective response of patients according to mutation type is summarized in Table 3 and Table S4. The majority (33/40) had rare V600 mutations, namely V600R, V600A, V600D, and V600_K601_delinsE. Of those, 42% showed either complete or partial response after target therapy. Mutations found at codon 600 were. V600_K601_delinsE was present in melanoma from only two cases treated with target therapy. One underwent target therapy as first‐line treatment, obtaining partial response; the other had progressive disease both after first‐line immune therapy and after second‐line target therapy.

**TABLE 3:** Response to target therapy according to rare BRAF mutation type.

Mutation	*N*	CR	PR	SD	PD	NA
T599dup	1	0	1	0	0	0
T599I	2	0	0	0	0	2
V600A	1	0	0	0	0	1
V600D	5	1	0	1	2	1
V600R	25	1	5	4	3	12
V600_K601delinsE	2	0	1	0	1	0
K601E	4	1	1	0	1	1
	40	3	8	5	7	17

Abbreviations: CR, complete response; *N*, number of cases; PD, disease progression; PR, partial response; SD, stable disease.

K601E, the most frequent variant outside codon 600 in our cohort, was identified in 56 individuals, of whom only 4 received first‐line target therapy, with treatment response information available for 3. Of those, 1 had a complete response, and 1 had a partial response, whereas 1 showed disease progression. At last, a patient with BRAF T599dup mutant melanoma, who progressed after immune therapy, was treated with target therapy, obtaining a partial response. Given the low frequency of each mutation, we were unable to perform formal statistical analyses for a variant‐per‐variant comparison. Therefore, these mutations were shortlisted for functional assessment by in‐silico modeling.

### 3.4 Overall Survival and Progression‐Free Survival

**FIGURE 2:**
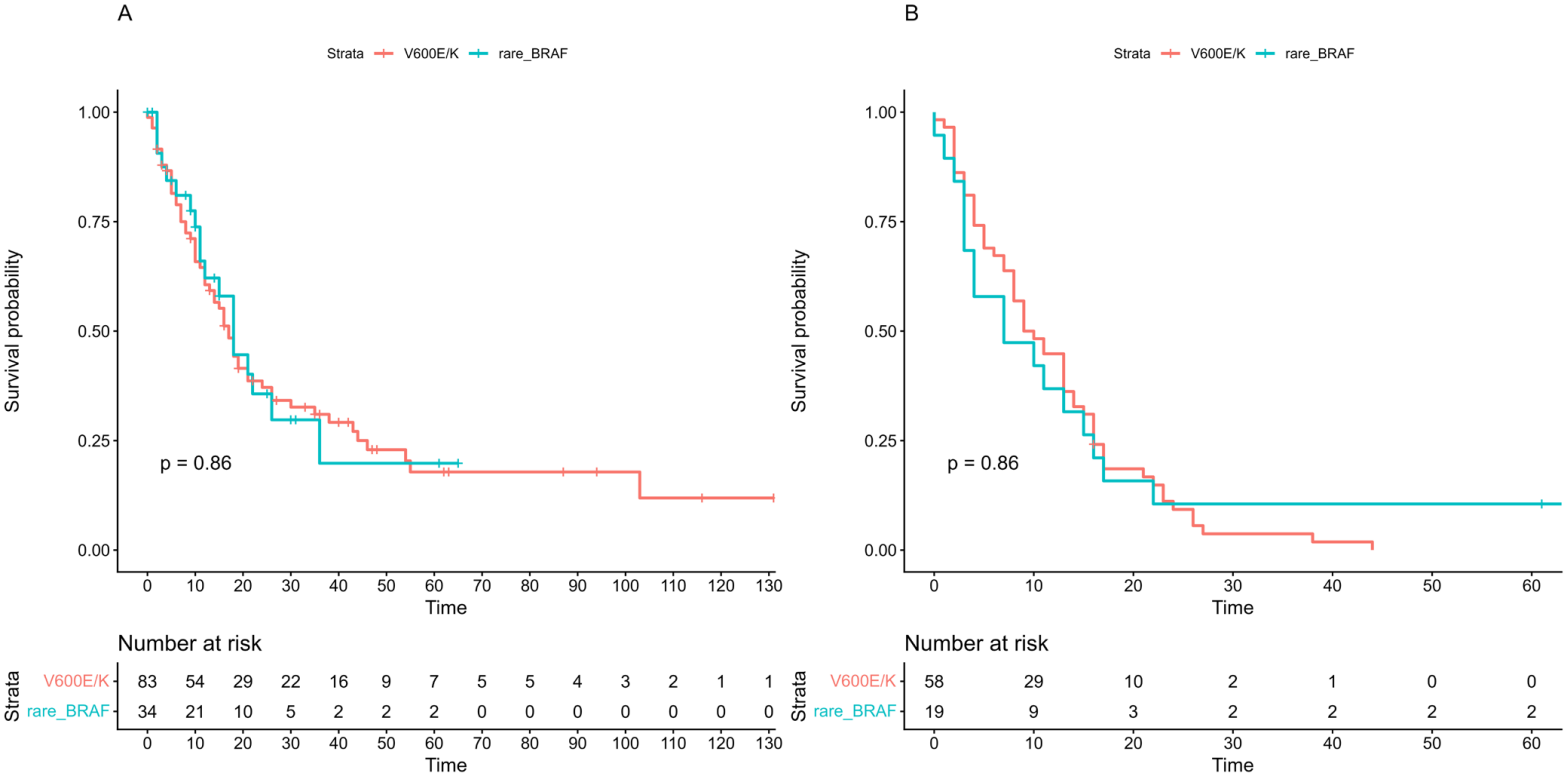
Overall survival (A) and progression‐free survival (B) in rare_BRAF and V600E/K undergoing first line or second line treatment with target therapy. The Kaplan–Meier curves show similar overall survival in both groups. Vertical bars indicate censored cases. Risk tables showing number of individuals at risk in each group are displayed below each plot.

**FIGURE 3:**
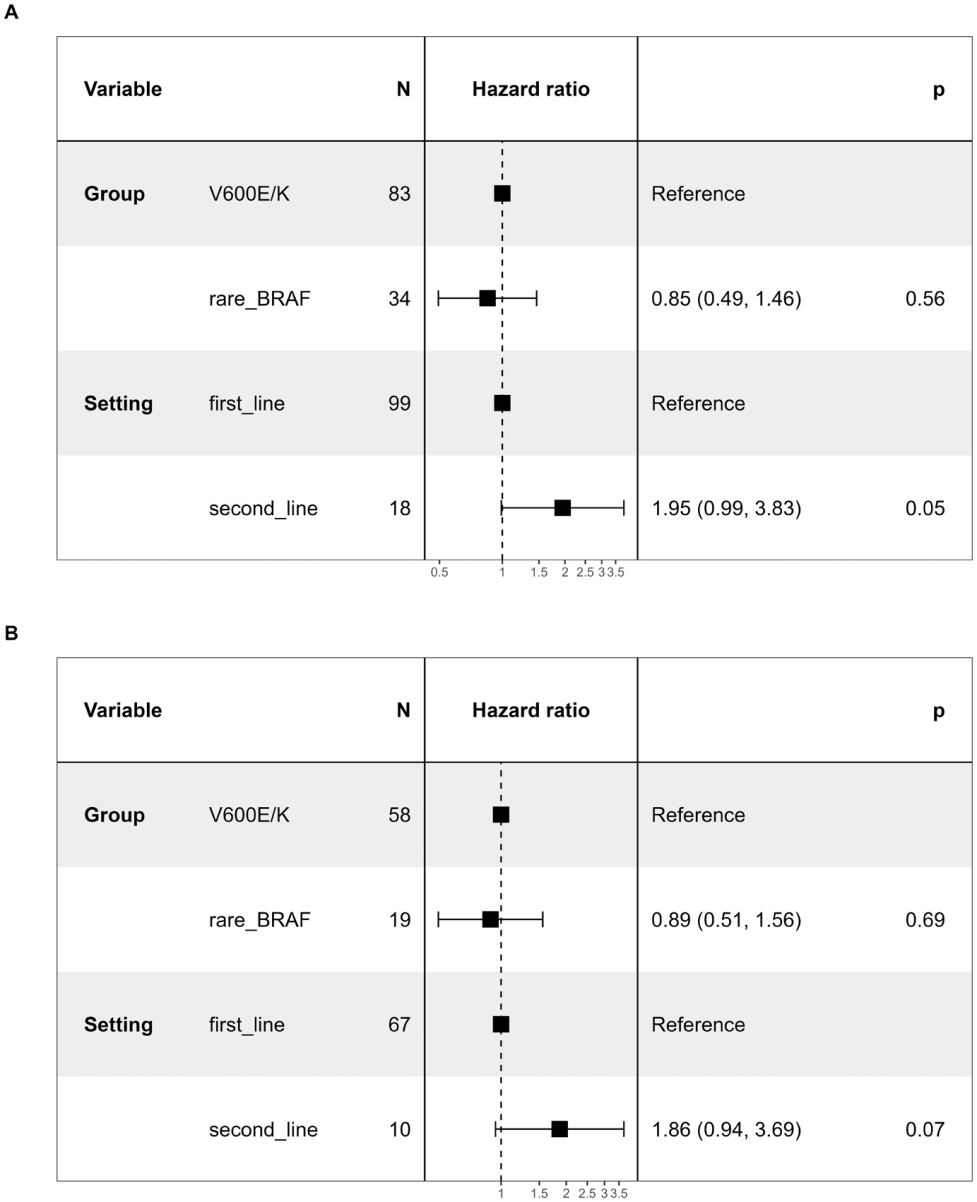
Multivariable survival analysis in cases treated with target therapy. The forest plots displaying the cox‐proportional hazard regression model adjusted for therapy setting. (A) Adjusted hazard ratios for Overall survival; (B) adjusted hazard ratios for progression‐free survival. 95% confidence intervals of hazard ratios are shown in parentheses.

In the cohort subset treated with target therapy, median OS was 11 months (95% CI = 5.0–21.3) in the rare BRAF and 15 months (95% CI = 6–33) in the V600E/K group. Median progression‐free survival (PFS) was 10 months (95% CI = 3.8–15.3) and 10.5 months (95% CI = 4–20.5) in the rare BRAF and V600E/K groups, respectively. We found no significant association between BRAF somatic status and either OS (HR = 0.96, 95% CI = 0.57–1.61, *p* = 0.88) or PFS (HR = 0.95, 95% CI = 0.55–1.65, *p* = 0.87), as also shown by the Kaplan Meier curves (Figure 2). Considering that the uneven distribution of cases treated with target therapy as first or second line treatment between the two groups could be a potential confounding factor, we also performed a multivariate Cox proportional hazard regression model which considered therapy setting as covariate, obtaining overlapping results (Figure 3).

### 3.5 Response to Therapy and Survival in Cases Treated With Immunotherapy

One hundred eight cases (70 rare_BRAF and 38 V600E/K) were treated with immune checkpoint inhibitors. Although ORR did not significantly differ (31% and 30%, *p* > 0.05) (Table S5), OS and PFS seemed longer in the non‐V600E/K compared to the V600E/K group (HR = 0.31, 95% CI = 0.17–0.56, *p* < 0.01 and HR = 0.38, 95% CI = 0.19–0.78, *p* < 0.01, respectively). However, this difference was only due to an enrichment of cases treated in the first‐line setting in the non‐V600E/K group. Indeed, multivariate Cox‐proportional hazard regression models corrected for therapy setting and age showed no significant difference in adjusted hazard ratios both for OS (HR = 0.69, 95% CI = 0.28–1.71, *p* = 0.42) and PFS (HR = 0.59, 95% CI = 0.21–1.67, *p* = 0.3) (Figures S3 and S4).

### 3.6 Modeling

**FIGURE 4:**
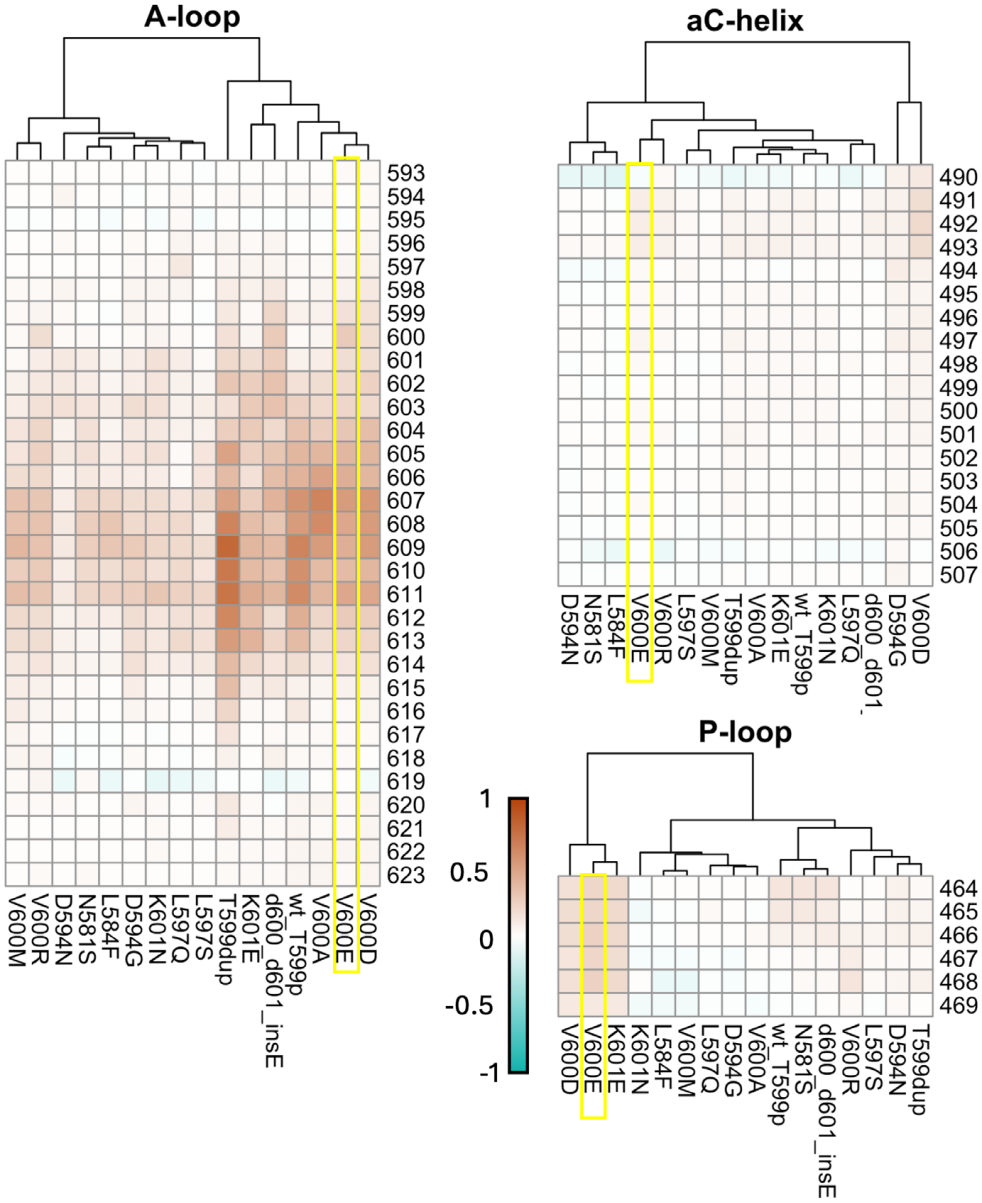
Graphical representation of the results from molecular dynamics simulations. The RMSF heatmaps illustrate the fluctuations observed during the simulations, normalized to the wt BRAF protein. The *x*‐axis represents the BRAF mutants, with each column corresponding to a different mutant, whereas the *y*‐axis indicates the BRAF residues analyzed in the simulation. Yellow boxes were used to highlight the RMSF fluctuations of the most characterized BRAF mutant, V600E, in each panel. The residues corresponding to the three regions that undergo the most significant conformational changes (A‐loop, P‐loop, and aC‐helix) are divided into three separate panels and clustered according to RMSF profiles. Legend scale: RMSF values of the mutants were normalized against those of the wt, resulting in a range from −1 to 1. Positive values indicate increased flexibility in the mutant, with values close to 1 reflecting a substantial increase in residue fluctuations compared to the wt. Conversely, negative values represent reduced oscillation and flexibility, with values close to −1 indicating residues more rigid in the mutant.

The level of destabilization of the A‐loop, αC helix, and P‐loop (Figure 1) of BRAF in terms of Root Mean Square Fluctuations (RMSF) predicted by the microsecond scale molecular dynamics simulations is shown in Figure 4 and Figure S5. For each variant, the RMSF profiles were calculated in triplicates compared to the values obtained for BRAF WT.

Higher RMSF values indicate greater local conformational freedom and, thus, a greater propensity for destabilization and conformational change.

BRAF variants analyzed in this study can be grouped as follows: those insisting on codon 600, that is, V600E/D/A/M/R; variants close to V600, that is, K601E/N, V600_K601delinsE, T599_V600insT, T599dup, and variants mapping before or at the beginning of the activation loop, namely N581S, L584F, D594G/N, L597Q/S.

We first observed that glutamic acid in position 600, V600E, affects the flexibility analogously to the phosphomimetic, WT BRAF‐T599p leading to destabilization of the region and spontaneous conformational change toward open BRAF conformations (Figure 4, Figures S5 and S6). This result is consistent with previous studies of the V600E mutant, resulting in a hyperactivation of the kinase domain and hyperphosphorylation of MEK (Roskoski 2010; Wan et al. 2004).

Second, we categorized BRAF variants according to their flexibility in the A‐loop, where we observed the greatest fluctuations (Figure 4, Figures S5 and S6).

The first cluster of variants encompasses those that introduce a net negative charge (aspartic and glutamic acid) at positions 599, 600, or 601. We found that they cluster with the WT BRAF‐T599p. This suggests that adding a negative charge in this area is sufficient to destabilize the loop and initiate the BRAF conformational switch to its active form. It is noteworthy that the V600A variant also clusters with negatively charged variants, even though it does not contribute any net charge to the destabilization of the inactive conformation. This suggests that alanine introduces greater flexibility due to its limited size and hindrance.

A similar destabilizing effect is observed in the case of T599dup (T599_V600insT), which may elongate the loop and destabilize the closed state, preventing the V600 loop from fitting optimally into the hydrophobic cavity (Figure S6).

The second cluster of variants includes those with bulky residues at position 600, either basic (histidine, lysine, and arginine) or hydrophobic (phenylalanine, methionine) (Figure 4, Figures S5 and S6). These variants are still destabilizing, although to a lower extent, most likely due to unproductive or suboptimal interactions with residues of the hydrophobic cluster.

Similarly, variants affecting codon 601, that is, K601E, K601N, or V600_K601delinsE alter the length and/or charge pattern of the T599‐K601 region. This results in a less pronounced destabilization of A‐ and P‐loop and in a moderate increase in the propensity to adopt the open/active conformation (Figure 4, Figures S5 and S6). Non V600 variants found in melanoma from cases for whom response to target therapy was available belong either to the first (T599dup) or the second (K601E) cluster.

Finally, the effect of more distal variants, spanning residue 594–597, was found to be less evident. Although A‐loop variants influence the flexibility of the 600–612 segment, which is clearly destabilized by WT BRAF phosphorylation at position 599 (WT BRAF T599p) under physiological conditions, the degree of destabilization varies and is dependent on the particular variant. For instance, the replacement of aspartate 594 with the small, apolar glycine results in a moderate destabilization of the region, whereas the mutation in asparagine results in a much smaller destabilization. D594, part of the aspartate‐phenylalanine‐glycine DFG triad (DFG motif), is involved in the coordination of Mg ions and ATP binding. Therefore, its destabilization implies a loss of kinase activity of the resulting protein, and thus destabilization should be considered in this sense.

In conclusion, although no variant significantly destabilizes the αC‐helix, the P‐loop is slightly destabilized by the introduction of a net charge at positions 599 and 600 but is less affected by distal variants or those that reduce the overall charge of the region (Figure 4 and Figures S5 and S6).

## 4 Discussion

Target therapy with BRAF and MEK inhibitors, along with immunotherapy, is one of the pillars of melanoma systemic therapy. Treatment with BRAF and MEK inhibitors is approved for actionable BRAF mutations. However, solid data on actionability are only available for V600E and V600K mutations (Sondak et al. 2024; Swetter et al. 2024). Indeed, although other variants involving both codon 600 and other exon 15 codons have been reported, they are found at low frequency, and studies examining their effect on melanoma phenotype and response to therapy are scant and often provide conflicting results. Here, by collecting data from 19 Italian centers, we found rare BRAF variants in approximately 2% of melanoma cases, accounting for the different techniques used. When NGS—the gold standard technique for specificity and, most importantly, sensitivity—was applied, the detection rate ranged from 1.98% to 6.35% (Table S1 and Figure S1).

We did not observe any significant difference in response to therapy and prognosis in melanoma cases with rare somatic pathogenic BRAF variants compared to those with V600E and V600K variants. Overall, our results corroborate previous findings on the efficacy of targeted therapy in the treatment of melanoma harboring rare BRAF variants compared to V600E/K mutant melanoma (Girod et al. 2022; Menzer et al. 2019).

Indeed, Giraud et al. (Girod et al. 2022) showed sensitivity to targeted therapy in cases with melanomas harboring rare BRAF mutations encompassing codon 600; the same study, however, suggested that variants outside codon 600 might respond less well to systemic treatment with MAPKi but, as reported by the authors, this finding might have been biased due to the small sample size and the different distribution of the therapy setting in which MAPKi were administered. Our study provides results on aggregated variants, and the contribution of each different variant could not be determined due to the shallow frequency of most of these variants. Consequently, we were unable to either confirm or refute this hypothesis.

In parallel, some of the BRAF variants were analysed by molecular dynamics simulations to assess their effect on the protein structure and kinase activity. This approach showed that 599–601 site is a hotspot where variants can have a strong impact on BRAF activity, and that a BRAF variant that insists on those positions leads to destabilization of the A‐loop to varying degrees depending on the type of amino acid change. The insertion of an acidic amino acid (D or E) at position 600 or 601 results in increased flexibility that could promote physiological phosphorylation of BRAF, leading to the activation of the kinase domain. On the other hand, the effect of amino acid substitution with non‐negatively charged amino acids is much more variable. Those that are small and/or uncharged, such as alanine, as well as those that are large and positively charged or hydrophobic, also have a destabilizing effect, although less effective.

Moreover, the introduction of apolar/polar and small amino acids does not cause any destabilization of the A‐loop at all. In conclusion, the modeling allowed us to examine different “hot” regions of the protein as well as different amino acid changes. This emphasizes the importance of carefully considering and assessing the type of mutation, as it can yield different effects. For instance, specific mutations affecting codon 594 which were identified in our cohort result in impaired kinase activity, as reported in previous studies and confirmed by our modeling analysis. According to BRAF functional classification (Sahin and Klostergaard 2021; Wan et al. 2004), these mutations fall into class III and are considered not actionable by BRAF/MEKi.

This, however, did not influence our results on survival and response to therapy, as melanoma harboring these mutations did not belong to cases treated with targeted therapy.

Rare BRAF mutations were not associated with specific melanoma histotype or thickness. However, melanoma harboring these mutations is often diagnosed in older patients compared with those with V600E or V600K mutations, suggesting potential differences in melanoma initiation, development and/or progression.

Our study has multiple limitations: BRAF mutations of the majority of cases treated with target therapy are on or encompass codon 600. Therefore, results on survival and response to therapy may have been influenced by non‐E/K V600 mutations, thus potentially masking differences regarding specific mutations outside V600. Similarly, we cannot exclude that differences between V600E and V600K might be a confounder, but we chose to group them together, as further subsetting would have resulted in reduced sample size and number of events for survival analysis. Another limitation lies in the heterogeneity of molecular testing across centres. Initial use of V600‐targeted assays, followed by broader methods such as NGS or Sanger sequencing, may have led to under‐detection of some non‐V600 variants, potentially resulting in a conservative estimate of rare mutation prevalence. Moreover, the retrospective nature of this study and the extensive recruitment time span resulted in heterogeneity of treatment regimens and incomplete retrieval of clinical determinants related to therapy response and survival.

To conclude, our study shows non‐inferiority of target therapy response in the IMI cohort of patients with melanomas harboring rare BRAF mutations compared to those with V600E/K mutant melanoma. However, this finding, which is in line with what was previously reported about rare BRAF mutations affecting codon 600, does not provide additional insights on rare mutations outside codon 600 due to their low frequency and the small number of treated cases. Nevertheless, in‐silico modeling conducted on rare non‐V600 mutations supports previous literature on response to target therapy in rare BRAF mutant melanoma (Girod et al. 2022; Menzer et al. 2019), and may justify further investigation into the nature of rare non‐V600 BRAF mutations in melanoma.

Considering the actionability of specific rare mutations, BRAF sequencing outside V600 could be conducted to identify a greater number of melanoma cases eligible for target therapy. Further research, including in vitro studies of specific variants, will be essential to determine the role of each variant in melanoma behavior and sensitivity to existing therapies.

## Author Contributions

**Maria Chiara Scaini:** investigation, writing – review and editing, writing – original draft. **Bruna Dalmasso:** investigation, writing – review and editing, writing – original draft, formal analysis. **Monica Rodolfo:** investigation, writing – review and editing. **Elena Tamborini:** investigation, writing – review and editing. **Ilaria Mattavelli:** investigation, writing – review and editing. **Carlo Cota:** investigation, writing – review and editing. **Francesca Collina:** investigation, writing – review and editing. **Elisa Melucci:** investigation, writing – review and editing. **Gerardo Ferrara:** investigation, writing – review and editing. **Maurizio Lombardo:** investigation, writing – review and editing. **Andrea Carugno:** investigation, writing – review and editing. **Stefania Tommasi:** investigation, writing – review and editing. **Laura Cendron:** writing – original draft, investigation, writing – review and editing, formal analysis. **Gabriele Madonna:** investigation, writing – review and editing. **Laura Orgiano:** investigation, writing – review and editing. **Francesca Castiglione:** investigation, writing – review and editing. **Simona Sola:** investigation, writing – review and editing. **Riccardo Marconcini:** investigation, writing – review and editing. **Stefania Pellegrini:** investigation, writing – review and editing. **Enrica Teresa Tanda:** investigation, writing – review and editing. **Martina Ubaldi:** investigation, writing – review and editing. **Michele Guida:** investigation, writing – review and editing. **Roberta Depenni:** investigation, writing – review and editing. **Andrea Boutros:** investigation, writing – review and editing. **Alireza Jorkesh:** investigation, writing – review and editing. **Francesco Spagnolo:** investigation, writing – review and editing. **Paolo Antonio Ascierto:** investigation, writing – review and editing. **Paola Ghiorzo:** investigation, writing – review and editing, conceptualization, writing – original draft, funding acquisition. **Italian Melanoma Intergroup:** resources. **Daniela Massi:** investigation, writing – review and editing. **Matteo Gasparotto:** investigation, writing – review and editing. **Chiara Menin:** investigation, writing – review and editing, writing – original draft, conceptualization. **Cristina Pellegrini:** investigation, writing – review and editing. **Lorenza Pastorino:** conceptualization, investigation, formal analysis, writing – original draft, writing – review and editing. **Giuseppe Palmieri:** investigation, writing – review and editing. **Mario Mandalà:** investigation, writing – review and editing, conceptualization. **Pietro Quaglino:** investigation, writing – review and editing. **Edoardo Raposio:** investigation, writing – review and editing.

## Funding

Funded by the European Union—Next Generation EU—NRRP M6C2—Investment 2.1 Enhancement and strengthening of biomedical research in the NHS, PNRR‐MCNT2‐2023‐12378166, CUP C73C23000840006 to P.G., G.M., M.C.; Italian Ministry of Health, IRCCS Azienda Ospedaliera Metropolitana, RC to P.G.; Alleanza Contro il Cancro RCR‐2022‐23682293ACCORD ACC‐FAMMEL (P.G., C.M.); Italian Ministry of Education PRIN 2022, 20222MTTNX to PG; Italian Ministry of Health, RCR‐2021 to Alleanza Contro il Cancro Melanoma Working Group (M.R.); Italian Ministry of Health (RC 2022‐2024—Linea 2/1—PI Ascierto PA).

## Ethics Statement

CER Liguria 198/2023.

## Conflicts of Interest

The authors declare no conflicts of interest.

## Supporting information


**Table S1:** Type and frequency of rare BRAF and canonical V600E/K mutations in participating centers.
**Table S2:** Clinical and pathological characteristics of cases included in the study.
**Table S3:** Clinical characteristics of cases treated with target therapy according to BRAF mutation type.
**Table S4:** Response to therapy according to rare BRAF mutation in the target therapy‐treated subset.
**Table S5:** Clinical and pathological characteristics of cases treated with immune checkpoint inhibitors.
**Figure S1:** Type and frequency of rare BRAF mutations within the Italian Melanoma Intergroup (IMI) study cohort.
**Figure S2:** Number of cases analyzed by molecular techniques and their corresponding RARE BRAF mutation rates (%).
**Figure S3:** Kaplan–Meier curves showing overall survival (A) and progression‐free survival (B) in rare_BRAF and V600E/K cases who underwent first line or second line treatment with immune checkpoint inhibitors.
**Figure S4:** Multivariable survival analysis in cases treated with immune checkpoint inhibitors.
**Figure S5:** RMSF graphs of the kinase domain residues of all the BRAF mutants analyzed in this study.
**Figure S6:** Structures used in the simulations: each mutant model is represented in cartoon view and colored according to the scale used in the heatmaps (indicating normalized fluctuations).

## Data Availability

The data that support the findings of this study are available from the corresponding author upon reasonable request.
