# Supplementary material for: Dissecting the Spectrum of Rare BRAF Mutations in Melanoma: A Nation‐Wide Study by the Italian Melanoma Intergroup (IMI)
Source: Pigment Cell Melanoma Res. 2026 Apr 19;39:e70087. doi: 10.1111/pcmr.70087 (PMC13092998; doi:10.1111/pcmr.70087)
Supplement: Supplementary file 1 — Table S1: Type and frequency of rare BRAF and canonical V600E/K mutations in participating centers. Table S2: Clinical and pathological characteristics of cases included in the study. Table S3: Clinical characteristics of cases treated with target therapy according to BRAF mutation type. Table S4: Response to therapy according to rare BRAF mutation in the target therapy‐treated subset. Table S5: Clinical and pathological characteristics of cases treated with immune checkpoint inhibitors. Figure S1: Type and frequency of rare BRAF mutations within the Italian Melanoma Intergroup (IMI) study cohort. Figure S2: Number of cases analyzed by molecular techniques and their corresponding RARE BRAF mutation rates (%). Figure S3: Kaplan–Meier curves showing overall survival (A) and progression‐free survival (B) in rare_BRAF and V600E/K cases who underwent first line or second line treatment with immune checkpoint inhibitors. Figure S4: Multivariable survival analysis in cases treated with immune checkpoint inhibitors. Figure S5: RMSF graphs of the kinase domain residues of all the BRAF mutants analyzed in this study. Figure S6: Structures used in the simulations: each mutant model is represented in cartoon view and colored according to the scale used in the heatmaps (indicating normalized fluctuations). [file PCMR-39-0-s001.docx]

**Supplementary Table 1**. **Type and frequency of rare BRAF and canonical V600E/K mutations in participating centers.**

| **Center ID** | **Cohort** | **Rare_BRAF** | | **V600E/K** | | **Techniques used (N)** |
| --- | --- | --- | --- | --- | --- | --- |
|  | **N** | **N** | **%** | **N** | **%** |  |
| IMI_1 | 252 | 4 | 2.38% | 102 | 40.48% | EP (171); NGS (81); |
| IMI_2 | 398 | 13 | 3.27% | 185 | 46.48% | NGS (398) |
| IMI_3 | 760 | 6 | 0.79% | 330 | 43.42% | NGS (115); PNA (645); |
| IMI_4 | 322 | 6 | 1.86% | 134 | 41.61% | NGS (32); RT (170); S (120); |
| IMI_5 | 736 | 9 | 1.36% | 349 | 47.42% | MT (305); NGS (12); RT (419); |
| IMI_6 | 1027 | 30 | 3.02% | 405 | 39.44% | NGS (257); S (770); |
| IMI_7 | 284 | 4 | 1.41% | 111 | 39.08% | EP (210); NGS (10); PY (64); |
| IMI_8 | 235 | 3 | 1.28% | 118 | 50.21% | RT (159); VL (76); |
| IMI_9 | 3123 | 53 | 1.76% | 1486 | 47.58% | NGS (2753); RT (1); S (369); |
| IMI_10 | 404 | 8 | 1.98% | 160 | 39.60% | NGS (404) |
| IMI_11 | 200 | 3 | 2.50% | 91 | 45.50% | NGS (147); RT (53); |
| IMI_12 | 1469 | 37 | 2.52% | 635 | 43.23% | NGS (520); RT (567); S (382) |
| IMI_13 | 969 | 29 | 3.41% | 456 | 47.06% | PY (145); RT (499); S (325); |
| IMI_14 | 189 | 12 | 6.35% | 98 | 51.85% | NGS (189) |
| IMI_15 | 271 | 5 | 1.85% | 120 | 44.28% | NGS (13); MT (146); PY (3); RT (109); |
| IMI_16 | 891 | 18 | 2.02% | 374 | 41.98% | NGS (891) |
| IMI_17 | 516 | 9 | 1.74% | 240 | 46.51% | NGS (189); PY (188); S (139); |
| IMI_18 | 1793 | 5 | 0.31% | 644 | 40.28% | EP (489); MT (1110); NGS (194); |
| IMI_19 | 242 | 4 | 1.65% | 93 | 38.43% | PY (242) |
|  |  |  |  |  |  |  |
| **Total** | **14081** | **258** | **1,83%** | **6131** | **43,54%** |  |

RT=RealTime PCR (only V600-); EP=RT-EasyPGX® only V600-); PNA=Peptide Nucleic Acid- ­RT-LAMP (only V600-); S=Sanger Sequencing; MT = Matrix-Assisted Laser Desorption/Ionization – Time of Flight-Mass Spectrometry (MALDI-TOF); PY=Pyrosequencing; VL= BRAF 600/601 StripAssay®; N= number of cases

| **Supplementary Table 2. Clinical and pathological characteristics of cases included in the study** | | | | |
| --- | --- | --- | --- | --- |
| **Variable** | **N** | **V600E/K**, N = 150 | **rare_BRAF**, N = 258^1^ | **p-value**^3^ |
| **Age at diagnosis^1^** | 359 | 59.0 (49.0, 68.0) | 65.5 (54.0, 75.0) | <0.001 |
| Missing |  | 17 | 32 |  |
| **Sex^2^** | 408 |  |  | 0.5 |
| F |  | 55 / 150 (37%) | 86 / 258 (33%) |  |
| M |  | 95 / 150 (63%) | 172 / 258 (67%) |  |
| **Histotype^2^** | 214 |  |  | 0.5 |
| SSM |  | 28 / 57 (49%) | 83 / 157 (53%) |  |
| NM |  | 25 / 57 (44%) | 67 / 157 (43%) |  |
| LMM |  | 0 / 57 (0%) | 1 / 157 (0.6%) |  |
| ALM |  | 0 / 57 (0%) | 2 / 157 (1.3%) |  |
| Other |  | 4 / 57 (7.0%) | 4 / 157 (2.5%) |  |
| Missing |  | 93 | 101 |  |
| **Death rate^2^** | 348 |  |  | <0.001 |
| Alive |  | 51 / 119 (43%) | 159 / 229 (69%) |  |
| Dead |  | 68 / 119 (57%) | 70 / 229 (31%) |  |
| Missing |  | 31 | 29 |  |
| **Breslow^1^** | 213 | 3.6 (2.1, 5.9) | 3.4 (1.5, 5.0) | 0.3 |
| Missing |  | 111 | 84 |  |
| ^1^Median (IQR). ^2^n / N (%). ^3^Wilcoxon rank sum test; Pearson's Chi-squared test; Fisher's exact test  Abbreviations: F=female, M=male, SSM= superficial spreading melanoma, NM= nodular melanoma, LMM= lentigo maligna melanoma, ALM= acral lentiginous melanoma | | | | |

| **Supplementary Table 3. Clinical characteristics of cases treated with target therapy according to BRAF mutation type** | | | | | | | | | |
| --- | --- | --- | --- | --- | --- | --- | --- | --- | --- |
| **Variable** | **N** | **rare_BRAF** N = 40*^1^* | **V600E** N = 77*^1^* | **V600K** N = 12*^1^* | **p-value^3^** | **q-value^4^** | ***Pairwise comparisons^5^*** | | |
|  |  |  |  |  |  |  | V600E vs rare_BRAF | V600K vs rare_BRAF | V600K vs V600E |
| **Age at diagnosis (years)^1^** | 121 | 61.5 (53.0, 73.5) | 59.0 (47.0, 66.0) | 75.0 (61.0, 81.0) | 0.011 | 0.095 | 0.464 | 0.176 | 0.011 |
| Missing |  | 0 | 7 | 1 |  |  |  |  |  |
| **Age at therapy start (years)^1^** | 125 | 64.5 (56.0, 74.0) | 61.0 (48.0, 69.0) | 81.0 (68.0, 82.0) | 0.002 | 0.021 | 0.270 | 0.087 | 0.003 |
| Missing |  | 2 | 2 | 0 |  |  |  |  |  |
| **Sex^2^** | 129 |  |  |  | 0.5 | >0.9 | 1.000 | 1.000 | 0.970 |
| F |  | 12 / 40 (30%) | 27 / 77 (35%) | 2 / 12 (17%) |  |  |  |  |  |
| M |  | 28 / 40 (70%) | 50 / 77 (65%) | 10 / 12 (83%) |  |  |  |  |  |
| **Histotype^2^** | 49 |  |  |  | 0.2 | >0.9 | 0.314 | 1.000 | 1.000 |
| SSM |  | 12 / 19 (63%) | 8 / 26 (31%) | 1 / 4 (25%) |  |  |  |  |  |
| NM |  | 6 / 19 (32%) | 14 / 26 (54%) | 3 / 4 (75%) |  |  |  |  |  |
| LMM |  | 0 / 19 (0%) | 0 / 26 (0%) | 0 / 4 (0%) |  |  |  |  |  |
| ALM |  | 0 / 19 (0%) | 0 / 26 (0%) | 0 / 4 (0%) |  |  |  |  |  |
| Other |  | 1 / 19 (5.3%) | 4 / 26 (15%) | 0 / 4 (0%) |  |  |  |  |  |
| Missing |  | 21 | 51 | 8 |  |  |  |  |  |
| **OS status^2^** | 126 |  |  |  | 0.062 | 0.6 | 0.069 | 1.000 | 1.000 |
| ALIVE |  | 19 / 39 (49%) | 20 / 75 (27%) | 4 / 12 (33%) |  |  |  |  |  |
| DEAD |  | 20 / 39 (51%) | 55 / 75 (73%) | 8 / 12 (67%) |  |  |  |  |  |
| Missing |  | 1 | 2 | 0 |  |  |  |  |  |
| **Best response - 1st/2nd line^2^** | 108 |  |  |  | 0.3 | >0.9 | 1.000 | 0.572 | 0.576 |
| CR |  | 3 / 23 (13%) | 13 / 74 (18%) | 2 / 11 (18%) |  |  |  |  |  |
| PR |  | 8 / 23 (35%) | 34 / 74 (46%) | 7 / 11 (64%) |  |  |  |  |  |
| SD |  | 5 / 23 (22%) | 8 / 74 (11%) | 2 / 11 (18%) |  |  |  |  |  |
| PD |  | 7 / 23 (30%) | 19 / 74 (26%) | 0 / 11 (0%) |  |  |  |  |  |
| Missing |  | 17 | 3 | 1 |  |  |  |  |  |
| **ORR – 1st/2nd line^2^** | 108 | 11 / 23 (48%) | 47 / 74 (64%) | 9 / 11 (82%) | 0.2 | >0.9 | 0.677 | 0.229 | 0.953 |
| Missing |  | 17 | 3 | 1 |  |  |  |  |  |
| **median OS** | 120 | 11.0 (5.0, 22.0) | 16.0 (5.0, 33.0) | 12.0 (8.0, 34.5) | 0.6 | >0.9 | 0.827 | 1.000 | 1.000 |
| Missing |  | 5 | 4 | 0 |  |  |  |  |  |
| **median PFS** | 112 | 10.0 (3.0, 16.0) | 10.5 (4.0, 21.0) | 11.0 (7.0, 26.0) | 0.7 | >0.9 | 1.000 | 1.000 | 1.000 |
| Missing |  | 9 | 7 | 1 |  |  |  |  |  |
| ^1^Median (Q1, Q3); ^2^n / N (%);^3^Kruskal-Wallis rank sum test; Fisher's exact test. ^4^Bonferroni correction for multiple testing.^5^Bonferroni-adjusted p-values for pairwise Fisher Exact test or Wilcoxon Wilcoxon Rank Sum Test. Abbreviations: F=female, M=male, NM= nodular melanoma, SSM= superficial spreading melanoma, CR= complete response, PR= partial response, SD= stable disease, PD= progressive disease, ORR= overall response rate, OS= overall survival, PFS= progression-free survival | | | | | | | | | |

| **Supplementaty Table 4. Response to therapy according to rare BRAF mutation in the target therapy-treated subset** |
| --- |

| **BRAF mutation(s)** |  | **I line** | **best response** | **II line** | **best response** |
| --- | --- | --- | --- | --- | --- |
| T599dup |  | Immuno | PD | Target | PR |
| T599I | K601N | Target |  | NO |  |
| T599I | K601N | Target |  |  |  |
| V600_K601delinsE |  | Target | PR | NO |  |
| V600_K601delinsE |  | Immuno | PD | Target | PD |
| V600A |  | Target |  |  |  |
| V600D |  | Target | PD | Carbo-Tax | PD |
| V600D |  | Target | SD | Paclitaxel | PD |
| V600D |  | Target |  | NO |  |
| V600D |  | Target | CR | NO |  |
| V600D |  | Immuno | PD | Target | PD |
| V600R |  | Target |  |  |  |
| V600R |  | Target | PD |  |  |
| V600R |  | Target | SD | Tem | PD |
| V600R |  | Target |  |  |  |
| V600R |  | Target | PD | Target |  |
| V600R |  | Target | SD | Target | SD |
| V600R |  | Target | PR | Immuno | PD |
| V600R |  | Target | CR | NO |  |
| V600R |  | Target | SD | Immuno |  |
| V600R |  | Target |  | NO |  |
| V600R |  | Target |  | NO |  |
| V600R |  | Target |  | NO |  |
| V600R |  | Target |  | NO |  |
| V600R |  | Target |  | NO |  |
| V600R |  | Target |  | NO |  |
| V600R |  | Target |  | Immuno | PD |
| V600R | V600M | Target | PR | Immuno | PD |
| V600R |  | Target |  | NO |  |
| V600R |  | Target |  |  |  |
| V600R |  | Immuno | PD | Target | PD |
| V600R |  | Immuno | PD | Target |  |
| V600R |  | Immuno | PD | Target | SD |
| V600R |  | Immuno | PD | Target | PR |
| V600R |  | Immuno | PD | Target | PR |
| V600R |  | Immuno | PR | Target | PR |
| K601E |  | Target | CR |  |  |
| K601E |  | Target |  | Immuno |  |
| K601E |  | Target | PD | NO |  |
| K601E |  | Target | PR | Immuno | PR |
| Abbreviations: Tem: Temozolomide; Carbo-Tax= carboplatinum- paclitaxel. NO= no therapy | | | | | |

| **Supplementary Table 5. Clinical and pathological characteristics of cases treated with immune checkpoint inhibitors** | | | | |
| --- | --- | --- | --- | --- |
| **Variable** | **N** | **V600E/K** N = 38 | **rare_BRAF** N = 70 | **p-value^3^** |
| **Age_any setting (years)^1^** | 102 | 60.5 (54.0, 69.5) | 68.5 (57.0, 77.0) | 0.030 |
| Missing |  | 2 | 4 |  |
| **Sex^2^** | 108 |  |  | 0.9 |
| F |  | 13 / 38 (34%) | 23 / 70 (33%) |  |
| M |  | 25 / 38 (66%) | 47 / 70 (67%) |  |
| **Histotype^2^** | 63 |  |  | 0.3 |
| SSM |  | 6 / 19 (32%) | 21 / 44 (48%) |  |
| NM |  | 11 / 19 (58%) | 21 / 44 (48%) |  |
| LMM |  | 0 / 19 (0%) | 1 / 44 (2.3%) |  |
| ALM |  | 0 / 19 (0%) | 0 / 44 (0%) |  |
| Other |  | 2 / 19 (11%) | 1 / 44 (2.3%) |  |
| Missing |  | 19 | 26 |  |
| **OS status^2^** | 108 |  |  | <0.001 |
| ALIVE |  | 9 / 35 (26%) | 43 / 67 (64%) |  |
| DEAD |  | 26 / 35 (74%) | 24 / 67 (36%) |  |
| Missing |  | 3 |  |  |
| **Best response^2^** | 78 |  |  | 0.2 |
| CR |  | 1 / 33 (3.0%) | 7 / 45 (16%) |  |
| PR |  | 9 / 33 (27%) | 7 / 45 (16%) |  |
| SD |  | 8 / 33 (24%) | 13 / 45 (29%) |  |
| PD |  | 15 / 33 (45%) | 18 / 45 (40%) |  |
| Missing |  | 5 | 25 |  |
| **ORR^2^** | 78 | 10 / 33 (30%) | 14 / 45 (31%) | >0.9 |
| Missing |  | 5 | 25 |  |
| ^1^Median (IQR). ^2^n / N (%).^3^Wilcoxon rank sum test; Pearson's Chi-squared test; Fisher's exact test  Abbreviations: F=female, M=male, NM= nodular melanoma, SSM= superficial spreading melanoma, LLM=lentigo maligna melanoma, ALM=acral lentiginous melanoma, CR= complete response, PR= partial response, SD= stable disease, PD= progression of disease, ORR= overall response rate, OS= overall survival, PFS= progression-free survival | | | | |


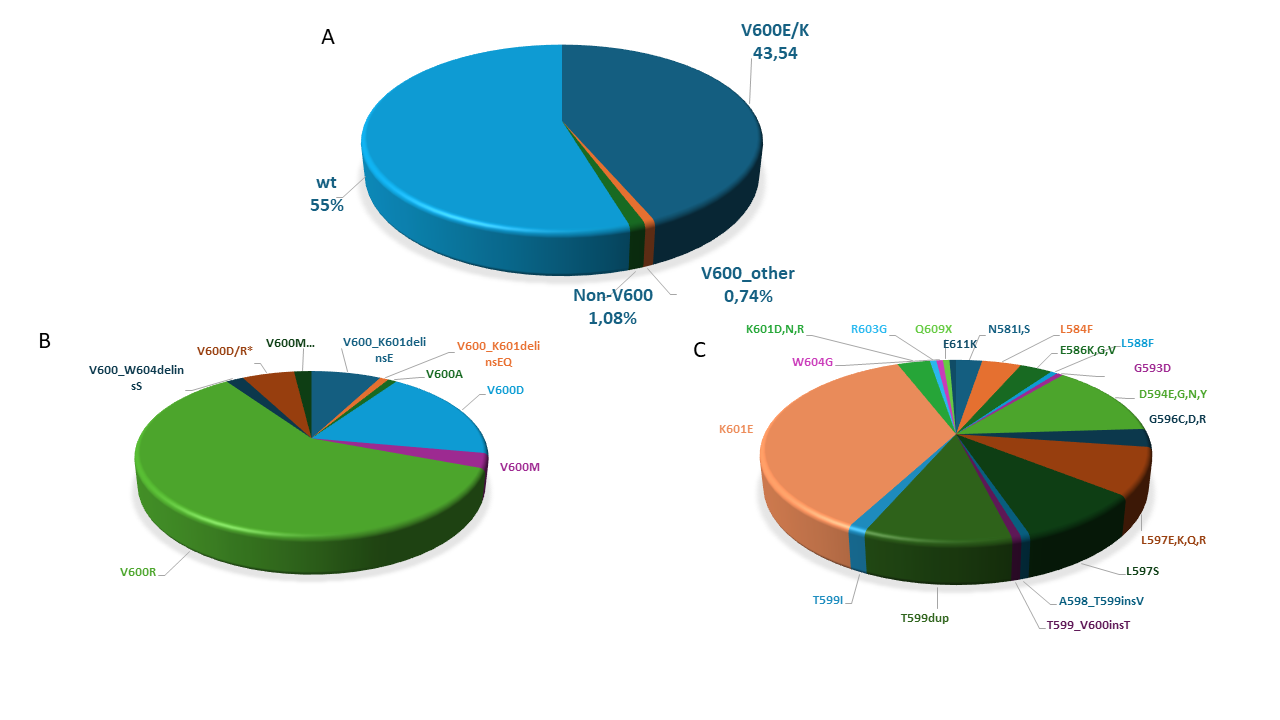


**Supplementary Figure 1**.


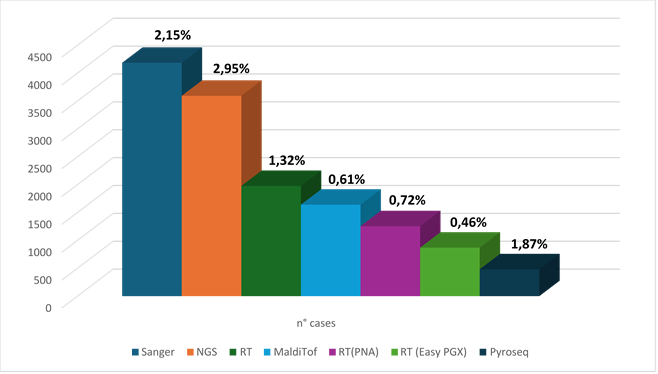


**Supplementary Figure 2.**

**
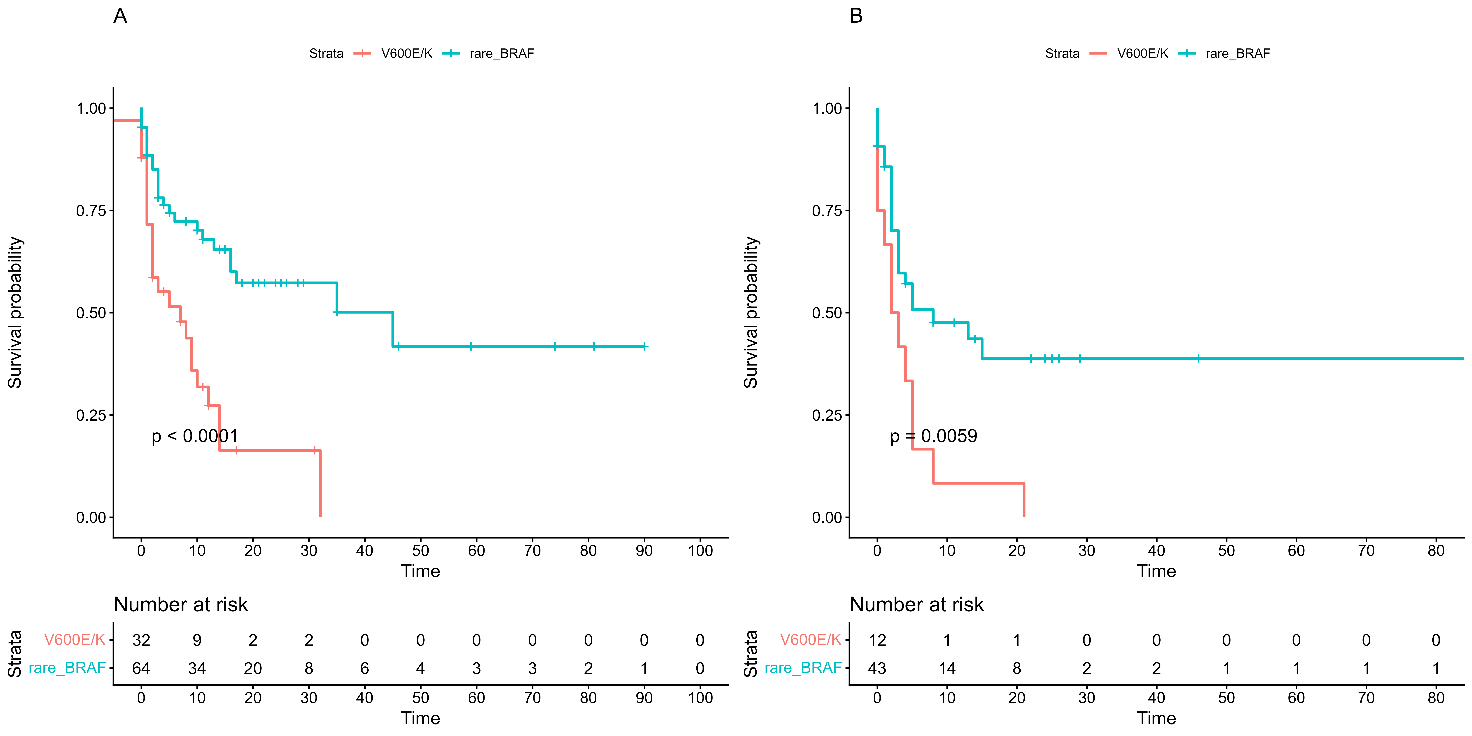
**

**Supplementary Figure 3**.


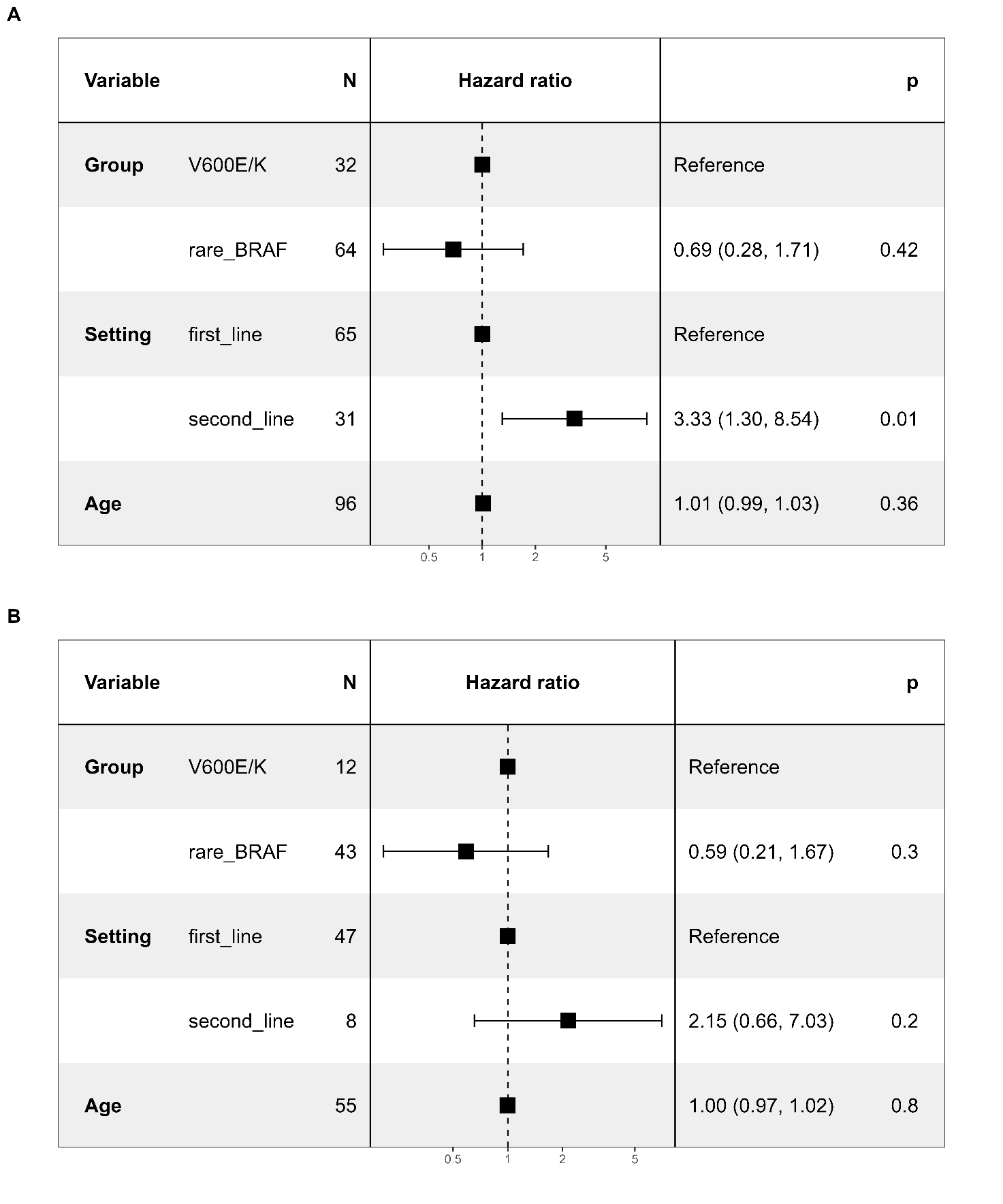


**Supplementary Figure 4.**


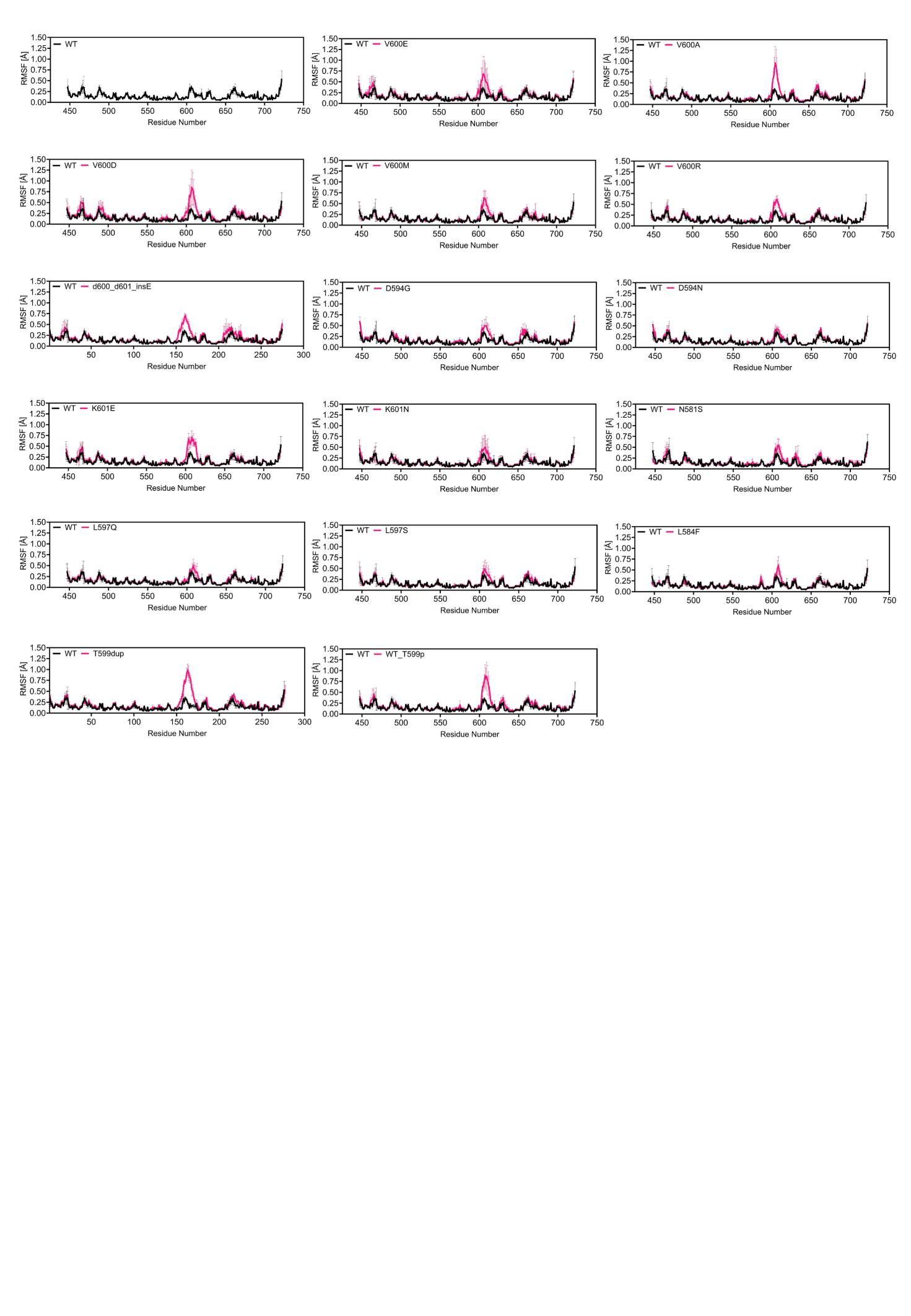


**Supplementary Figure 5.**


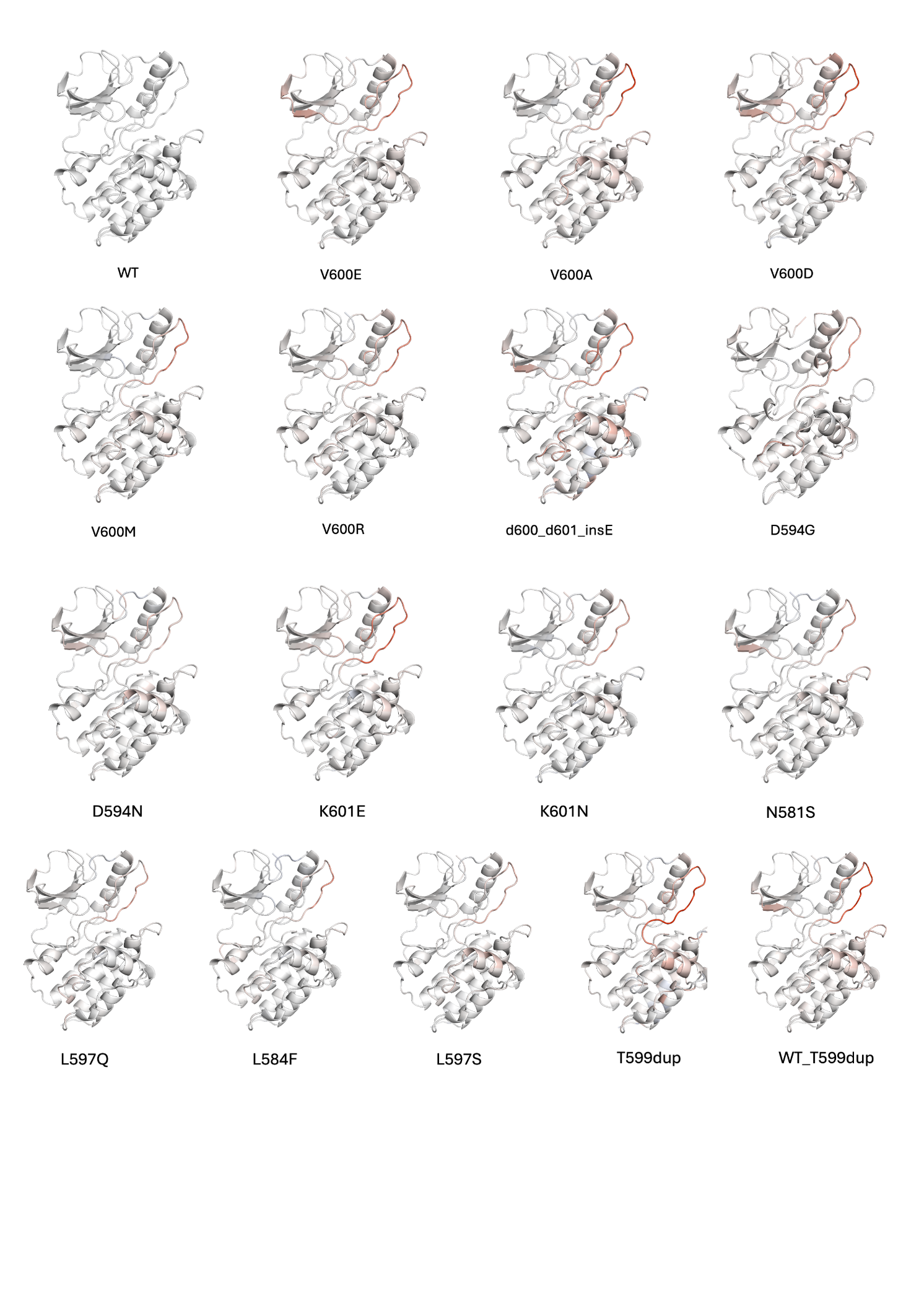


**Supplementary Figure 6.**
